# Perioperative management of neurosurgical patients receiving chronic anticoagulation therapy

**DOI:** 10.3389/fphar.2014.00064

**Published:** 2014-04-08

**Authors:** Arman Zakaryan

**Affiliations:** Department of Neurosurgery, Yerevan M. Heratsi State Medical UniversityYerevan, Armenia

**Keywords:** antiplatelet therapy, anticoagulation, neurosurgery, perioperative complications

In all neurosurgical cases associated with anticoagulation/antiplatelet therapy, perioperative antithrombotic management of patients is complicated due to increased risks of intra- and/or postoperative bleeding or thromboembolic complications. The potential risk of thrombosis may increase when anticoagulation/antiplatelet therapy is stopped perioperatively. On the other hand, perioperative hemorrhage may occur more often when anticoagulation/antiplatelet therapy is continued.

In general, the contraindications to anticoagulation/antiplatelet therapy include:

- Traumatic brain injury
- Craniotomy
- Hemorrhagic stroke
- Brain tumor
- Uncontrollable hypertension
- Preexisting coagulopathies and/or advanced hepatic or renal disease
- Other clinical situations.

In certain circumstances increased risk of thrombosis in a previously placed vascular stent (particularly, drug-emitting stents), deep vein thrombosis with a history of pulmonary embolism, neuroendovascular procedures, acute cardiovascular insufficiency, and a risk of acute thrombosis, therapy with anticoagulants and antiplatelet medications is crucial. In recent years the number of patients, receiving chronic anticoagulant and antiplatelet therapy increases consistently both among adult and pediatric patients (Gentilomo et al., 2011; Enomoto et al., 2014).

Venous thromboembolism is a significant risk in neurosurgical patients which is explained by the long operating times of many procedures, prolonged bed rest, immobility related to motor deficits (e.g., in spinal cord injuries or stroke patients), as well as release of significant amounts of brain tissue thromboplastin during surgery and trauma, diuretic therapy used to reduce cerebral edema, intravascular volume losses following subarachnoid hemorrhage, and cerebral salt wasting, etc.

Even though, prophylactic therapy with anticoagulants and antiplatelet drugs is effective in reducing the risk of thromboembolism, its use in elective neurosurgery remains controversial.

Perioperative antithrombotic therapy is performed more frequently and intensively during neuroendovascular procedures. Although anticoagulation may not increase the risk of aneurysm rupture, it would most likely increase the volume of hemorrhage when aneurysm rupture occurs and thus increase treatment-related morbidity and mortality.

Surgery for parenchymal lesions, such as brain tumors, when the surgery disrupts small vessels, is associated with a higher risk for hemorrhage.

Number of studies found no higher risk in patients treated with heparin or oral anticoagulation when the prothombine time/International Normalized Ratio is monitored closely (Altschuler et al., 1990; Constantini et al., 2001). Other studies indicate that the incidence of postoperative hemorrhage is increased in patients undergoing brain tumor surgery and receiving heparin or enoxaparin (So et al., 1983; Dickinson et al., 1998). Rouine-Rapp and McDermott (2013) recommend stopping clopidogrel preoperatively and using a bridging therapy with Eptifibatide during surgery (Rouine-Rapp and McDermott, 2013). The beneficial effects of the drug were related to its beneficial pharmacologic profile: following intravenous administration, plasma concentrations are achieved within 5 min, maximum inhibition of platelets occurs within 15 min, and steady state concentrations are attained within 4–6 h. Platelet aggregation usually returns to normal within 4 h of discontinuing therapy (Rouine-Rapp and McDermott, 2013). However, the safety and efficacy of such bridging therapy needs to be evaluated in prospective properly designed studies in various groups of neurosurgical patients before it can be recommended for routine clinical use.

As a conclusion, the number of patients receiving chronic anticoagulation therapy is consistently increasing, and many of those patients undergo neurosurgical procedures. This problem needs further evaluation to optimize the treatment approaches to these patients. In patients receiving anticoagulation/antiplatelet therapy who are scheduled for intracranial surgery, the benefits of such therapy should be weighed against the associated risks.

## Conflict of interest statement

The authors declare that the research was conducted in the absence of any commercial or financial relationships that could be construed as a potential conflict of interest.
